# Predictors of loss due to pharmaceutical fraud: evidence from the U.S.

**DOI:** 10.1186/s12962-022-00337-4

**Published:** 2022-02-12

**Authors:** Yuriy Timofeyev, Susan A. Hayes, Mihajlo B. Jakovljevic

**Affiliations:** 1 HSE University, Moscow, Russia; 2 College of Science, Health and Pharmacy, Roosevelt University, Chicago, IL USA; 3 Institute of Comparative Economic Studies, Hosei University, Tokyo, Japan; 4 Department of Global Health Economics and Policy, Faculty of Medical Sciences, University of Kragujevac, Kragujevac, Serbia

**Keywords:** Pharmaceutical fraud, Health care, Hierarchical linear model, Profiling, US

## Abstract

**Background:**

Globally and in the U.S. in particular, pharmaceutical fraud account for a large number out of all crimes in health care, which result into severe costs to the society. The Academy of Managed Care Pharmacists (Fraud, waste, and abuse in prescription drug benefits. 2019. Posted May 20. https://www.amcp.org/policy-advocacy/policy-advocacy-focus-areas/where-we-stand-position-statements/fraud-waste-and-abuse-prescription-drug-benefits.) estimate that pharmacy fraud is 1% of costs, therefore estimating that pharmacy fraud costs at $3.5 billion, given that pharmacy costs are $358 billion (Statista. Prescription drug expenditure in the United States from 1960 to 2020. 2021. https://www.statista.com/statistics/184914/prescription-drug-expenditures-in-the-us-since-1960/).

**Aim:**

This exploratory study aims to demonstrate a fraudster’s profile as well as to estimate average consequences in terms of costs and identify the loss predictors’ hierarchy in the pharmaceutical industry in the U.S.

**Materials and methods:**

Data from the Corporate Prosecution Registry and mixed-effects models are utilized for this purpose. The dataset covers years 2001–2020 and 75 cases, falling into one of the following broad sub-categories: misbranding, counterfeit, off-label use of drugs/deceptive marketing; violation of the Food, Drug and Cosmetic Act.

**Results:**

The main factors positively associated with loss due to pharmaceutical fraud are: (i) duration of , and (ii) the scheme and scheme being executed at a U.S. public company. Surprisingly, presence of collusion negatively and significantly effects the cost. Potential factors include: (a) principal perpetrator being a white American and/or male, and (b) number of employees at individual and organizational level respectively.

**Conclusion:**

This study empirically justifies considering loss, due to pharmaceutical fraud, from a multi-level perspective. Identified profiles of a typical fraudster helped to elaborate on specific practical recommendations aimed at pharmaceutical fraud prevention in the U.S.

## Introduction

Health care fraud in the United States costs between $100 billion and $300 billion [1]. The level of spending for fraud will only increase as the population in the United States ages. In 2017, Medicare, the largest program for health care in the United States, spent $702 billion. By 2028, Medicare’s expenditure is estimated to increase to $1.5 trillion, primarily due to an aging population and increases in the costs of health care services [2]. These assessments are largely aligned with the health spending forecasts for the US [3] and other major Emerging Markets up to 2025 [4] and 2030 [5].

Globally and in the U.S. in particular, pharmaceutical fraud account for a large number out of all crimes in health care, which result into severe costs to the society. The Academy of Managed Care Pharmacists [6] estimate that pharmacy fraud is 1% of costs, therefore estimating that pharmacy fraud costs at $3.5 billion, given that pharmacy costs are $358 billion [7].

One of the most comprehensive analyses of pharmaceutical fraud was performed by John Braithwaite [8] in 1984 and today remains a seminal analysis of fraud committed by pharmaceutical companies. Braithwaite interviewed 131 C-suite managers of world-wide pharmaceutical companies. This research covered topics, such as bribery, drug safety and unsafe manufacturing process, anti-trust, incentives for therapy initiation and financial abuses. Almost every company Braithwaite interviewed had some brush with illegal behavior and Braithwaite (p. 308), states that:

“*to my amazement, two American executives interviewed held the position of ‘vice-president responsible for going to jail’. The companies whose very mission it was to make drugs that were supposedly aimed at making people healthier realized that there had to be one senior person accountable when regulators caught up with them for their illegal behavior and demanded a ‘head for the chopping block’.*”

Braithwaite laid out a comprehensive three step strategy regulation, rehabilitation, and restitution as interventions for industry reform.

In 2014, Dukes, Braithwaite and Maloney [9] sought to update Braithwaite initial seminal study. The author’s intent was not to reproduce the original study of the pharmaceutical industry but to understand if, a generation later, Braithwaite’s roadmap for regulation, rehabilitation and restitution had been realized. More than three decades later, the most significant change is that the monetary scale of recent fraud is even greater, allowing for inflation. Bribery increased in the last generation in major economies, such as China, India and Italy (p. 213), while in the United States price hikes to the “highest level the market will bear (p. 224)” resulted in illegal re-importation from developing countries and counterfeiting of medications. Rather than locking up hundreds of executives from the pharmaceutical industry, the greatest potential for cleaning up the pharmaceutical industry lies not in increasing punishment, but in increasing detection (p. 283). Corporate crime enforcement that is ‘internationally entrepreneurial,’ that is financially beneficial to pharmaceutical manufacturers through competition and innovation remains a neglected approach to pharmaceutical fraud.

It is clear that this ideal of pharmaceutical industry self-regulation has not been realized. Recent headlines include a physician and pharmaceutical sales representative involved in false billing with $2.2 million in restitution [10], the Italian units of Bayer and Novartis were charged with operating a scheme to cheat the regional public health service in Lombardy with five hospitals agreeing to pay €200,000 as an agreed penalty [11] and a Florida man who pled guilty to one charge of conspiracy to commit health care fraud in $1 billion fraud case whereby he and several others defrauded pharmacy benefit managers out of nearly $175 million [12].

While the financial costs of health care fraud generate headlines, the impact on human health is of greater concern. For example, in 2013, drug manufacturer Abbott Laboratories Inc. paid $1.5 billion to resolve allegations that it illegally promoted a drug to treat agitation and aggression in elderly dementia patients and schizophrenia patients, when neither of these uses are approved as safe and effective by the FDA [13]. Such off-label use of medication resulted in exacerbated medical conditions for elderly patients by not promptly treating dementia and schizophrenia.

These headline cases, combined with growth in spending in health care and, more importantly, health care fraud has prompted this study. This exploratory study aims to demonstrate a pharmaceutical fraudsters’ profile, as well as to estimate average consequences in terms of costs and identify the loss predictors’ hierarchy in the pharmaceutical industry in the United States. Our primary research questions are as follows. First, what are the key features of pharmaceutical fraud in general and in the US specifically? Second, which factors, individual or organizational, play a dominant role when predicting costs due to pharmaceutical fraud? Third, which individual factors are associated with higher loss due to pharmaceutical fraud? Fourth, which organizational factors are associated with higher loss due to pharmaceutical fraud? Fifth, what can be done in order to reduce the cost of pharmaceutical fraud?

The paper is organized as follows. After the introduction, we review definitions of pharmaceutical fraud and the relevant studies on loss due to (pharmaceutical) fraud. Then, we introduce data and methods. Next, we present and discuss the results. In the last section, we discuss our results in the context of previous studies and point to the further research directions.

## Definition of pharmaceutical fraud in the United States

Pharmaceutical fraud is a subset of health care fraud. Fraud is often an elusive concept to define [14]. In general, fraud is defined as the wrongful or criminal deception intended to result in financial or personal gain. Fraud is therefore often difficult to prove because we do not know if the fraudster *intended* to deceive or if a simple error occurred. However, for purposes of this study, the cases we review as pharmaceutical fraud as fraud are ones in which the perpetrator was: (a) involved in deception about pharmacy products or services, and (b) convicted of a violation of the Food, Drugs and Cosmetic Act. The fraudsters that we profile in this study have already been convicted of fraud, therefore, we are not assuming fraud occurred but rather by virtue that they were convicted of fraud, we are concluding that these perpetrators acted fraudulently.

The Food, Drug and Cosmetic Act (FDCA) [15] has a long history in the United States, having been passed in 1938 and establishing the Food and Drug Administration (FDA) as a regulatory agency ([16], p. 138). The FDCA has been amended many times to reflect changes in the respective Food, Drug and Cosmetic industries. The Federal Food, Drug, and Cosmetic Act and subsequent amending statutes are codified into Title 21 Chapter 9 of the United States Code and is a Federal law. Chapter V of the Act relates to Drugs, and within the Chapter, each Section is devoted to the crime associated with violations of the Act. For example, Section 501 concerns itself with Adulterated drugs and devices, Section 502 with Misbranded drugs and devices, and so on. For each of the cases we reviewed, we categorized each case into the following four sub-categories according to the Sections (and crimes) associated with the FDCA ([16], p. 138): misbranding (Section 501), counterfeiting [Section 801(a)], off-label use (Section 301) and professional practice considerations (Section 353) [17]. Off-label use of pharmaceuticals is controversial. Many community physicians prescribe off-label use of medications [18]. However, it is illegal for a pharmaceutical company to *promote* the use of off-label medication for indications which have not been approved by the FDA.

## Predictors of loss due to pharmaceutical fraud

In addition to the seminal works mentioned above by Braithwaite, there have been many studies related to predicting pharmacy fraud. Many of these studies focus on using data mining techniques to detect fraudulent cases. Konijn and Kowalczyk [19] presented a novel approach to finding pharmacy fraud using “outlier-ness” (claims with anomalous characteristics compared to standard deviation from the mean) in claims data. A more recent article published by Liu et al. [20], focused on statistical methodology and a visual means (cluster optics) and machine learning to detect pharmacy fraud [20].

In regard to profiling perpetrators of health care fraud, little has been written on this topic. Kennedy et al. attempted to better understand the crime of pharmaceutical counterfeiting through developing a crime script for pharmaceutical counterfeiting that describes key acts, scenes, actors, activities, and enforcement conditions. Occupational counterfeiters leverage their position as a health care provider to abuse patient trust and conceal their deviant acts [21]. Qureshi et al. [22], in a similar study, profiled the crimes (but not the perpetrator) of violators of the FDCA. The authors highlighted that many of the largest pharmaceutical corporations have been implicated in health care fraud cases, sometimes more than once. The authors predicted that with expansion of government health care, investigations of pharmaceutical manufacturers will continue to result in substantial financial recoveries. Their findings raised concern that despite these recoveries, industrywide changes in the way pharmaceutical corporations conduct marketing activities were needed [22]. Timofeyev and Jakovljevic [23] conducted a study that determined within the mental health setting, typical fraudster’s profile is defined as a 53-year old male psychiatrist. In addition, Medicaid, the existence of collusion, and fraudster’s age are associated with the fraud loss. Review of the literature concludes that profiling of health care fraudsters in an attempt to detect these crimes has not been extensively conducted.

While not directly related to healthcare fraud, perpetrator profiling research has been conducted in other aspects of white-collar crime, specifically economic crime (theft, embezzlement, deception, accounting fraud/manipulation, kickbacks, insider trading, money laundering and counterfeiting). Bussman and Werle [24] conducted a victimization survey and reported that perpetrators where highly educated, high social status, males over the age of 40. A more recent study conducted in England and Wales and Norway also reports that perpetrators of bribery were male and middle aged [25]. Similar to this study, Andersen and Button, as well as the ACFE’s Report to the Nation, reinforce that the profiles of white-collar perpetrators are predominately male, middle-aged (approximately 40 years old) and highly educated. In the 2020 ACFE’s Report to the Nations, bachelor degree perpetrators were likely to be the most predominate group, with average losses of USD 175,000 per incident, with a postgraduate degree causing a median loss of USD 200,000 [26].

Peltier-Rivest [27] studied three cases involving pharmaceutical companies (Eli Lilly and Company, Pfizer, Inc. and Johnson & Johnson) where pharmaceutical fraud had occurred against the framework of the fraud diamond. The purpose of exploring the fraud diamond was to illustrate that not only are the traditional motivators for fraud evident in pharmaceutical fraud, as depicted by the fraud triangle as casual factors (financial pressure, an ethical rationalization and a perceived opportunity). The fraud diamond adds a fourth side to the fraud triangle and considers an individual’s *capability*: personal traits and abilities that play a major role in whether fraud may actually occur even with the presence of the other three elements. Using the fraud diamond theory, Peltier-Rivest [27] demonstrates that the following strategies are effective at preventing pharmaceutical corruption:

*“Offering employee assistance programs and revising performance goals tied to sales or stock prices; using transformational leadership; offering and certifying employee training on key company policies and anti-bribery legislations; using open-door policies and anonymous reporting mechanisms; assessing corruption risks associated with doing business in the world’s poorest countries and contracting with third-party agents; implementing proper anti-corruption controls such as segregating the research funding function from the sales division; and detecting common corruption schemes, such as fictitious marketing agreements with off-shore entities and sham contracts with doctors, through the analysis of relevant red flags.”*

While limited, the studies that have been performed, show that perpetrators leverage their position to abuse patient trust, pharmaceutical companies are repeat offenders and age, low-income patients and collusion with other health care providers are flags of potential health care fraud.

## Data

### Data collection procedure

We utilize data from the Corporate Prosecution Registry. 70 relevant U.S. cases dated 2001–2020, falling into the category of “*Fraud—healthcare*” and “*FDCA/Pharma*”, are selected for the initial sample. Following the Registry’s protocol, data on nine additional recent cases, dated 2020, we collected from open sources, such as, e.g., the U.S. Department of Justice (DoJ) website. Cases without a guilty plea or trial conviction were removed. Also, we removed cases with zero cost, calculated as a sum of leaving us “Total payment” and “Additional regulatory fine or payment”. This left a sample of 75 observations. President George W. Bush (January 20, 2001–January 20, 2009) was in office during 15 out of 75 (20%) cases; President Barack Obama (January 20, 2009–January 20, 2017) was in office for 39 (52%) of the cases and President Donald Trump (January 20, 2017–January 20, 2021) was in office for 21 (28%) of the cases.^1^ Socio-demographic data on the principal perpetrator’ characteristics are manually collected from the other sources like the U.S. Securities and Exchange Commission. These data include age, gender and race. Respective pharmaceutical companies’ characteristics are collected from *Thompson Reuters* database. Characteristics include number of employees, annual revenue and years in business at the year the crime was revealed. Our dependent variable and continuous case- and company-specific variables were subject to logarithmic transformations in order to make their distributions close to normal. For the purpose of robustness check, we replaced missing values by average values of the respective variables.

## Methods

We follow the approach of Timofeyev [28] and Timofeyev and Jakovljevic [23] to analyze factors of fraud-related costs. We use hierarchical linear models: (i) to identify the importance of each level’s predictors affecting the size of cost; and (ii) to reveal predictors associated with the size of cost due to pharmaceutical fraud. The appropriate models are presented below.

We solve the first task by analyzing the intra-class correlations with a help of the following empty multilevel model, which decomposes the variance in size of cost.

1 $$lnY_{ijk} = \, \gamma_{000} + \, \upsilon_{00k} + \, \delta_{0jk} + \, \varepsilon_{ijk}$$

In Eq. (1), *lnY*_*ijk*_ is the dependent variable, namely, the natural logarithm of dollar loss caused by the perpetrator’s participation in fraudulent activities in case *i* in year *j* in state *k*; *γ*_*000*_ is the grand mean of costs caused by the perpetrator’s participation in fraudulent activities. The sources of cross-state variation in losses, which cause particular states to deviate from the grand mean, are contained in *υ*_*00k*_. Similarly, *δ*_*0jk*_ contains sources of variation among years. Finally, *ε*_*ijk*_ captures inter-case differences. *ω*_*00*_*, τ*_*00*_*,* and *σ*^*2*^ represent the variances of case-, year-, and state-level sources respectively. To argue that all three levels are important, all of these variance components have to be statistically significant and account for a sufficiently large intra-class correlation [29].^2^ The second task related to identifying the losses’ predictors is resolved by the means of the full hierarchical linear model (2) with different specifications of the vector *X* [30].

2 $$lnY_{ijk} = \, \gamma_{000} + X_{ijk} + \, \upsilon_{00k} + \, \delta_{0jk} + \, \varepsilon_{ijk}$$

Our models include perpetrator-, case-3 and company-specific variables. We performed statistical using Stata 16.0 (Stata Corp.). All models were fitted via maximum likelihood.

In addition, for the purpose of a robustness check, we use jurisdiction-year and jurisdiction-period grouping in Eqs. (1) and (2). Next, as the number of observations in the sample is relatively small and it may result in unreliable parameter estimation when estimate parameters of model (2) directly. In order to make full use of the original data and improve the accuracy of estimation, we adopt the Bootstrap method [31] by repeating sampling 500 times to estimate parameters in model (2). Bootstrap is a feasible and effective method to deal with small sample data [32].

Finally, we have replaced missing observations in the independent variables using multiple imputation [33]. This is only acceptable, if data is missing completely at random (MCAR) that is indicated by a statistically insignificant value of the Little’s MCAR test (> 0.1).^4^

## Results

### Descriptive statistics

Table 1 represents the main variables description from Corporate Prosecution Registry. Table 2 provides summary statistics for the basic sample.

**Table 1:** Variables’ description

Variable	Type	Description	
Company	String	Name of company	
Date	Date (YYYY-MM-DD)	For deferred and non-prosecution agreements, this field reflects the date of the agreement. For acquittals, dismissals, plea agreements, and trial convictions this field reflects the date of the judgement or dismissal. For declinations, this field reflects the date of the declination	
Disposition_type	String	Describes how the dispute with an organization was resolved. Can take on the following values: Acquittal, declination, dismissal, DP^a^, NP^b^, plea, trial conviction	
Jurisdiction	String	The U.S. Attorney’s Office(s) involved	
Primary_crime_code	String	Can take on the following values:	
		Health Care Fraud	These include prosecutions brought under 18 U.S.C. § 1347
		Pharmaceutical	These include prosecutions brought under the Federal Food, Drug, and Cosmetic Act (FDCA) as well as anti-kickback and other related claims involving pharmaceutical sales and branding
Total_payment	Integer	Sum of fine, forfeiture, restitution, etc. amounts in U.S. dollars	
Additional_regulatory_fine_or_payment	Integer	Sum in U.S. dollars	
US_public_company	Boolean	Indicates whether or not entity was a U.S. public company	

Source: Corporate Prosecution Registry

^a^DP means “Deferred Prosecution”. It is like corporate probation. If the company does what is in the DP agreement and does not get in trouble again, the company escapes any restitution or findings requirements. Many times the company has to employ a Corporate Compliance Officer that reports to the court if the charges are serious

^b^NP refers to “Non-Prosecution Order”, which is a little less than a DP. The company is ordered to do something, but there is no prosecution pending. So the company might pay a fine, agree to make a product using better standards, etc.

**Table 2:** Summary statistics for the basic sample

Variable	Obs	Mean	Std. Dev.	Min.	Max.
Cost	75	184 mln	473 mln	400	2800 mln
ln(cost)	75	15.097	3.800	5.992	21.753
Total_payment	75	140 mln	411 mln	0	2800 mln
Additional payment	66	49.8 mln	179 mln	0	900 mln
Duration	52	52.673	32.142	9	156
ln(duration)	52	3.749	0.716	2.197	5.050
Duration^a^	75	39.280	33.511	9	156
ln(duration)^a^	75	3.273	0.934	2.197	5.050
Collusion	74	0.108	0.313	0	1
Collusion^a^	75	0.107	0.311	0	1
in_biz	68	32.471	35.306	2	153
ln(in_biz)	68	2.971	1.043	0.693	5.030
ln(in_biz)^a^	75	2.971	0.993	0.693	5.030
n_employees	53	7897	22,372	1	109,208
ln(n_employees)	53	5.298	3.100	0	11.601
ln(n_employees)^a^	75	5.298	2.598	0	11.601
Annual_revenue	46	4120 bln	22,800 bln	83,711	153,000 bln
ln(revenue)	46	18.471	4.996	11.335	32.662
ln(revenue)^a^	75	18.471	3.896	11.335	32.662
U.S. public company	75	0.173	0.381	0	1
Age	56	53.214	9.816	35	84
Age^a^	75	53.214	8.463	35	84
Male	71	0.958	0.203	0	1
Male^a^	75	0.960	0.197	0	1
White	73	0.877	0.331	0	1
White^a^	75	0.880	0.327	0	1
Misbranding	65	0.446	0.501	0	1
Counterfeiture	66	0.106	0.310	0	1
Off_label_use	66	0.303	0.463	0	1
Pharm_practice_act	65	0.154	0.364	0	1

^a^Indicates the variables with multiple imputations

#### Perpetrators’ characteristics

The overwhelming majority of our perpetrators are males (68 out of 71, 96%), white (64 out of 73, 88%), in their early 50 s (N = 56; mean = 53.214; SD = 9.816; min = 35; max = 84).

#### Case-specific characteristics

Typically, the scheme lasted around 4.5 years (N = 52; mean = 53 months; SD = 32 months; min = 9; max = 156 months). More than one perpetrator was involved into eight out of 74 cases (11%). 29 out of 64 (45%) cases referred to misbranding. The remaining cases included: promoting drug for non-approved use; counterfeit drugs; off-label use of drugs; (use of) adulterated drugs; kickbacks for promoting drugs; conspiracy to distribute controlled drugs; illegal distribution of a new drug; illegally marketing/promoting drugs; compounding veterinarian meds; selling pain creams; failure to report on clinical studies for a new drug; failure to transmit information about a drug; distribution of drug for use other than Food and Drug Administration (FDA) indications.

#### Companies’ characteristics

13 out of 75 (17%) cases occurred in U.S. public companies. On average, there were around 7897 employees working at a company at the year the crime was revealed (N = 53; SD = 22,372; min = 1; max = 109,208). Average age of the company, at the year the crime was revealed, was 32 years (N = 68; SD = 35.306; min = 2; max = 153).

### Correlation and regression analysis

Table 3 demonstrates variables’ pairwise correlations. The following variables are correlated at 5%-level with cost due to pharmaceutical fraud: indicator for U.S. public company, company’s age, and indicator for off-label use of drugs.

**Table 3:** Cross-correlations

	Cost	Age	Male	White	Collusion	Duration	US_public	In_biz	N_employees	Revenue	Misbranding	Counterfeit	Off_label_use	Pharm_fraud_act
Cost	1													
Age	− 0.0281	1												
Male	0.0776	− 0.0364	1											
White	0.0893	0.1619	− 0.0800	1										
Collusion	− 0.1217	0.1436	− 0.1466	− 0.0018	1									
Duration	0.1927	− 0.1328	0.2210	− 0.1244	− 0.1268	1								
US_public	0.4153*	− 0.0562	0.0994	0.0656	− 0.1607	0.2642	1							
In_biz	0.4884*	0.2414	− 0.1115	0.1694	− 0.1706	0.0455	0.2112	1						
N_employees	0.1464	− 0.1126	0.0508	− 0.1531	− 0.1274	0.3868*	0.5079*	0.0935	1					
Annual revenue	0.0549	0.0301		0.0725	− 0.0639	0.5697*	− 0.0202	− 0.0174	0.0668	1				
Misbranding	− 0.1774	0.0211	0.1696	− 0.1451	− 0.0173	0.2679	− 0.0619	− 0.1354	− 0.1029	0.1734	1			
Counterfeiture	− 0.0422	− 0.0374	− 0.2233	− 0.2308	0.0394	0.1683	− 0.1706	0.0630	− 0.0219	0.6844*	− 0.1121	1		
Off_label_use	0.3274*	0.1014	0.1214	0.0917	− 0.0050	− 0.1281	0.3366*	0.1968	0.2459	− 0.1107	− 0.5984*	− 0.2271	1	
Pharm_fraud_act	− 0.1355	− 0.1528	− 0.2012	0.1409	− 0.0129	− 0.2280	− 0.2132	− 0.1448	− 0.1571	− 0.0805	− 0.2969*	− 0.1481	− 0.2843*	1

^*^p < 0.05

The estimates for Eq. (1) with 75 observations suggest that 50.7% of variance is explained by location. 7.5% of variance is explained by year. The remaining 41.8% is explained by individual and case-specific variables. Thus, three-level HLM is an appropriate technique.^5^

Table 4 represents estimates for Eq. (2). In columns 1–6 we use original data only, without fraud type dummies (columns 1–4) and with ones (columns 5 and 6). The major factors positively associated with loss due to pharmaceutical fraud include, first, duration of the scheme, and, second, scheme being executed at a U.S. public company. Surprisingly, presence of collusion (i.e., multiple perpetrators involved) negatively and significantly effects the cost. Potential factors, which can affect the cost due to pharmaceutical fraud, are: (a) principal perpetrator being a white American and/or male, and (b) company’s size (number of employees) at individual and organizational level respectively. Negative and statistically significant interaction terms of age and duration (columns 2, 4 and 8) imply that the longer the scheme is, the smaller the effect of age on the size of loss becomes: or, alternatively, the earlier the scheme is detected, the larger the effect of age on costs becomes. The estimates with multiple imputations for missing values (columns 7–12) support the initial estimates with the original data. The results with jurisdiction-year and jurisdiction-period grouping are qualitatively the same and are available upon request. Estimates of bootstrapping with 500 iterations, for each regression with more than 44 observations, support our initial results.

**Table 4:** Estimates for Eq. (2): multi-level model with state-year grouping

Variables	(1)	(2)	(3)	(4)	(5)	(6)	(7)	(8)	(9)	(10)	(11)	(12)
	ln(cost)	ln(cost)	ln(cost)	ln(cost)	ln(cost)	ln(cost)	ln(cost)	ln(cost)	ln(cost)	ln(cost)	ln(cost)	ln(cost)
Age	0.0285	0.821***	0.00322	0.655***	0.00868	− 0.0254	0.0140	0.795***	**− 0.00592**	0.0104	**0.0150**	**0.00894**
	(0.0243)	(0.229)	(0.0266)	(0.225)	(0.0213)	(0.0218)	(0.0259)	(0.229)	(0.0287)	(0.0205)	(0.0281)	(0.0299)
Male	− 2.377	− 2.780	− 0.896	− 0.915	− 5.000**	− 0.900	− 1.679	− 2.149	**− 1.153**	− 5.206**	− 2.791*	**− 2.645**
	(2.168)	(2.446)	(1.955)	(2.058)	(2.142)	(3.559)	(2.014)	(2.338)	(1.363)	(2.170)	(1.692)	(1.702)
White	1.857***	3.541***	1.672**	2.894***	0.763	0.574	1.669**	3.312***	1.551**	0.818	**0.359**	**0.386**
	(0.652)	(0.727)	(0.695)	(0.738)	(0.650)	(0.901)	(0.694)	(0.751)	(0.783)	(0.608)	(0.854)	(0.861)
Collusion	− 4.501***	− 4.616***	− 3.619***	− 3.892***	− 3.940***	− 4.164***	− 3.776***	− 4.081***	**− 0.409**	− 4.011***	**− 1.291**	**− 1.008**
	(0.740)	(0.622)	(0.867)	(0.728)	(0.727)	(0.581)	(0.869)	(0.735)	(0.820)	(0.682)	(0.847)	(0.917)
ln(duration)	1.724***	13.49***	1.130**	10.66***	2.279***	1.232**	1.642***	13.18***	1.019***	2.486***	1.303***	1.258***
	(0.536)	(3.377)	(0.568)	(3.285)	(0.500)	(0.577)	(0.501)	(3.375)	(0.314)	(0.494)	(0.327)	(0.333)
US public company	3.561***	2.718***	3.484***	3.304***	2.102***	1.320	3.450***	2.842***	2.607***	1.860***	2.616***	2.544***
	(0.722)	(0.770)	(0.659)	(0.668)	(0.707)	(0.809)	(0.685)	(0.743)	(0.768)	(0.711)	(0.741)	(0.747)
ln(Years in business)			0.526**	0.381*	0.298		0.476*	**0.325**		**0.282**		**0.243**
			(0.266)	(0.220)	(0.285)		(0.265)	(0.220)		(0.277)		(0.324)
ln(N of employees)						0.215			0.434***		0.239*	0.244*
						(0.161)			(0.131)		(0.137)	(0.137)
Age*ln(duration)		− 0.211***		− 0.173***				− 0.207***				
		(0.0609)		(0.0601)				(0.0611)				
Fraud type dummies included	No	No	No	No	Yes	Yes	No	No	No	Yes	Yes	Yes
Constant	7.961***	− 36.92***	8.085***	− 28.09**	9.406***	13.04***	6.869**	− 36.77***	9.252***	9.526***	11.55***	11.11***
	(3.066)	(13.39)	(2.968)	(12.91)	(3.005)	(2.987)	(2.923)	(13.34)	(2.269)	(2.976)	(2.530)	(2.577)
Log likelihood	− 95.943	− 91.782	− 87.429	− 84.102	− 70.930	− 60.483	− 94.582	− 90.790	− 166.936	− 75.252	− 139.255	− 138.993
LR test vs. linear model	0.0001	0.0001	0.0006	0.0017	0.0041	0.0073	0.0011	0.0024	0.0008	0.0009	0.0013	0.0062
Observations	44	44	41	41	38	33	44	44	75	40	65	65
Number of groups	18	18	16	16	14	13	18	18	27	16	25	25

Standard errors in parentheses. ***p < 0.01, **p < 0.05, *p < 0.1

Coefficients are bold, if multiple imputations are made for missing values of the respective variables

## Discussion and conclusion

Our findings suggest two factors that contribute to pharmaceutical fraud. First, the longer the scheme endures, the greater the fraud. This is consistent with Wells [34] that indicate the longer fraud goes on, the amounts involved grow over time, and the perpetrator becomes careless about concealing the fraud. Kalovya [35] further stated that organizations should strive to minimize duration of fraud, since the evidence suggests the longer the duration before discovery the larger are the losses. Błaszczyński et al. [36] also concluded in a study on auto loan fraud that the longer a fraud goes undetected, the greater the financial losses to the organization. Therefore, early intervention of pharmaceutical fraud is important to reduce the impact to victims. More importantly, since the pharmaceutical fraud in our study involved several schemes that impacted patients, the earlier the intervention, the less impact on patient health. Some of the schemes actually involved counterfeit drugs; off-label use of drugs; (use of) adulterated (containing unapproved ingredients) drugs which could be detrimental to long term patient health.

A second finding is that greater fraud was perpetrated in public companies. This is not surprising. Public companies are often bigger than privately held companies with the likelihood that the there is more financial resources available. A company with sales in the billions clearly has more ability to generate fraudulent activity than a small business enterprise, defined by the U.S. International Trade Commission as a company with sales revenue under $7 million [37]. However, Krishnan and Peytcheva [38] state that “*the risk of fraud as higher for family firms than for non-family firms, consistent with the predictions of entrenchment theory*”. Auditors are also less likely to make client acceptance recommendations for family firms. The strength of the audit committee moderates the family-firm effect, whereby auditors assess family firms with weak [Audit Committees (ACs)] to have the highest fraud risk and to be the least desirable audit clients. Krishnan and Peytcheva’s findings suggest that auditors perceive more severe agency conflicts to be present in family firms than in non-family firms, consistent with entrenchment theory, according to which family members may behave opportunistically to extract rents and potentially expropriate the firm’s resources at the expense of minority shareholders. Many of the cases we reviewed were perpetrated by single or small businesses, such as the cases involving conspiracy to distribute controlled drugs; illegal distribution of a new drug; illegally marketing/promoting drugs; compounding veterinarian meds; and selling pain creams.

Our findings also suggest that collusion (i.e., multiple perpetrators involved) negatively and significantly effects the cost of the fraud. We believe this may be resultant of more perpetrators that are involved, the likelihood increases of red flags being displayed by at least one of them causing earlier detection. It might be more difficult to keep undetected a collusion in pharmaceutical sector, especially in large companies. ACFE’s Report to the Nations on Occupational Fraud and Abuse [39], which showed that nearly half of the examined cases involved multiple perpetrators colluding with one another to commit fraud, and the greater the number of fraudsters involved, the higher losses tended to be.

Lastly, our study identified potential factors, which can affect the cost due to pharmaceutical fraud, are: (a) principal perpetrator being a white American male, and (b) company’s size (number of employees) at individual and organizational level respectively. Greater controls within publicly traded or large companies with these characteristics could potentially reduce fraud opportunities by greater controls, particularly controls which maintain the confidentiality of an informant, since management may be “in” on the scheme. These controls could be greater publication and acceptance by employees of tip or “hotlines” to report suspicious or fraudulent activity [40]. The 2018 ACFE Report to the Nations [41] study found that 63% of the victim organizations utilized tip hotlines. Of those who had hotlines, 46% of cases were detected by people that provided information that exposed fraud, compared with only 30% of cases detected not utilizing hotlines. In addition, losses at organizations who utilized hotlines were smaller: $100,000, compared to $200,000 for those organizations that did not utilize hotlines. Moreover, organization without hotlines were twice as likely to detect fraud by accident or external audit [41]. Ultimately, pharmaceutical companies vulnerable to fraud due to these risk predictors should have a complete assessment of all internal controls. Publicly-traded companies are required by the Sarbanes–Oxley Act of 2002 to use a recognized internal control framework in determining the proper controls to adopt, such as the framework developed by the Committee of Sponsoring Organizations (COSO) of the Treadeway Commission. The framework can also be useful to non-public companies in establishing a strong internal control program [40]

Among the limitations of this study we should mention, e.g., low number of observations. In addition, depending on the country, cost due to pharmaceutical fraud, measured as a court-assigned restitution, may vary significantly. We have also not addressed how much resource allocation, in terms of dollars spent in detection, prosecution and restitution *should* be spent in resolving health care. This is basically a philosophical decision of the cost of human health which was outside the scope of this research. In the future, we consider analyzing the data collected for other countries, which can allow cross-country comparisons. Also, it would be very insightful to assess the impact of perpetrators’ annual compensation growth ratio in the preceding years on cost. This can only be possible when more cases are included into the analysis.

## Data Availability

Data from the Corporate Prosecution Registry are available at https://corporate-prosecution-registry.com/.

## Footnotes

1 Presidential office-holding might play an important role since the President of the United States often sets the tone for actions of the Department of Justice charged with investigation and prosecution of white-collar crime.

2 Otherwise, simple OLS regression analysis with clustered standard errors is sufficient.

3 Length of the scheme in months and a dummy for cases with more than one perpetrator.

4 [Prob > chi-square] = 0.3428 for the following combination of variables: age, male, white, collusion, duration, number of employees, years in business, and fraud type dummies.

5 Estimates with jurisdiction-year and jurisdiction-period grouping suggest using two-level HLM because year and president’ period of rule become insignificant. While the first requires adding year-dummies and does not allow to converge some of our models, the grouping by period of president rule does not capture year-specific factors. Therefore, we do not use these models.
